# Bioactive glass BAG-S53P4 for the adjunctive treatment of chronic osteomyelitis of the long bones: an *in vitro* and prospective clinical study

**DOI:** 10.1186/1471-2334-13-584

**Published:** 2013-12-10

**Authors:** Lorenzo Drago, Delia Romanò, Elena De Vecchi, Christian Vassena, Nicola Logoluso, Roberto Mattina, Carlo Luca Romanò

**Affiliations:** 1 Laboratory of Clinical Chemistry and Microbiology, I.R.C.C.S. Galeazzi Orthopaedic Institute, Via R. Galeazzi 4, Milan 20161, Italy; 2 Department of Biomedical Sciences for Health, University of Milan, Via Mangiagalli 31, 20161, Milan, Italy; 3 Department of Reconstructive Surgery of Osteo-articular Infections C.R.I.O. Unit, I.R.C.C.S. Galeazzi Orthopaedic Institute, Milan 20161, Italy; 4 Department of Biomedical, Surgical and Odontoiatric Sciences, University of Milan, Via della Commenda 10, 20122, Milan, Italy

**Keywords:** Bone infections, *Staphylococcus aureus*, *Pseudomonas aeruginosa*, Bioactive glass

## Abstract

**Background:**

This study aimed to explore the in vitro antibacterial activity of the bioglass BAG S53P4 against multi-resistant microorganisms commonly involved in osteomyelitis and to evaluate its use in surgical adjunctive treatment of osteomyelitis.

**Methods:**

*In vitro* antibacterial activity of BAG-S53P4 against methicillin resistant *Staphylococcus aureus* and *Staphylococcus epidermidis, Pseudomonas aeruginosa* and *Acinetobacter baumannii* isolates was evaluated by means of time kill curves, with colony counts performed after 24, 48 and 72 hours of incubation. *In vivo* evaluation was performed by prospectively studying a cohort of 27 patients with a clinically and radiologically diagnosed osteomyelitis of the long bones in an observational study. Endpoints were the absence of infection recurrence/persistence at follow-up, no need for further surgery whenever during follow-up and absence of local or systemic side effects connected with the BAG use.

**Results:**

*In vitro* tests regarding the antibacterial activity of BAG S53P4 showed a marked bactericidal activity after 24 hrs against all the tested species. This activity continued in the subsequent 24 hrs and no growth was observed for all strains after 72 hrs. Results of the clinical study evidenced no signs of infection in 24 patients (88.9%) at the follow-up, while 2 subjects showed infection recurrence at 6 months from index operation and one more needed further surgical procedures. BAG-S53P4 was generally well tolerated.

**Conclusions:**

The *in vitro* and *in vivo* findings reinforce previous observations on the efficacy of BAG-S53P4 for the treatment of chronic osteomyelitis of the long bones, also in the presence of multi-resistant strains and in immunocompromised hosts, without relevant side effects and without the need for locally adding antibiotics.

**Trial registration:**

Deutschen Register Klinischer Studien (DRKS) unique identifier: DRKS00005332.

## Background

Besides haematogenous osteomyelitis, any kind of bone or soft tissue trauma or surgery where pathogens can reach the bone may cause infection. Osteomyelitis hallmarks consist of progressive bone destruction and the formation of sequestra. *Staphylococcus aureus,* other gram-positive and gram-negative bacilli are the pathogens most commonly involved [1]. Treatment usually includes surgical debridement of the necrotic and infected tissue that harbours bacteria and systemic antibiotics [2]. Complete infection eradication is still considered a challenging result, depending, among other variables, on the anatomo-pathological aspect of the disease and host type [3,4].

Moreover, various degrees of bone loss are frequently encountered in chronic osteomyelitis, due to the septic process *per se*, to the related inflammatory reactions and to the necessary surgical debridement, further complicating treatment and reconstruction of the affected segment. Bone defects are usually treated with local or free flaps, bone transport, or, more often, with local application of antibiotic-loaded polymethyl methacrylate (PMMA) beads, bone grafts or bone substitutes, aimed at filling the dead space while delivering antibacterial compounds locally [5-9]. Bioactive glasses (BAGs) have been recently shown to have antibacterial, osteoconductive and angiogenic properties [10-15]. The BAG-S53P4 composition (SiO_2_, Na_2_O_,_ CaO, P_2_O_5_) facilitates tissue growth by binding chemically the bone matrix and thereby promote the formation of new bone in the implanted area. In particular, antibacterial properties have been put in correlation with a local pH and osmotic pressure increase, since sodium and calcium ions and phosphorus salts make the environment hostile for bacterial adhesion and the subsequent proliferation of microorganisms that cause infection [15,16]. Despite the increasing amount of *in vitro* evidence concerning BAGs antibacterial properties, and the long term clinical use in treating chronically infected bone in cranio-maxillofacial indications [17,18], only two clinical studies, on a limited series of patients, have explored the potential use of BAGs for the treatment of chronic osteomyelitis so far in an orthopaedic setting [14,19].

Nonetheless BAGs seem to be particularly interesting in this application, since one version (S53P4, BonAlive®, Finland) is an approved medical device in Europe for the treatment of osteomyelitis, being the only biomaterial, to our knowledge, approved for local application for the treatment of bone infections without being pre-loaded with antibiotics or acting as an antibiotic carrier.

Aims of the present study were: i) to explore the *in vitro* antibacterial activity of BAG S53P4 against multi-resistant microorganisms commonly involved in osteomyelitis and ii) to report on the largest series published to date in the treatment of osteomyelitis with surgical debridement and local application of BAG S53P4.

## Methods

### *In vitro* study

#### Preparation and conditioning of bioglass

Tubes containing BAG-S53P4 granules (diameter of 500–800 μm) (BonAlive Biomaterials Ltd, Turku, Finland) or inert glass of similar size used as control (R1350 Iittala clear, Iittala, Finland) were prepared at a final concentration of 800 mg/mL and 400 mg/mL in Tryptic Soy Broth (TSB; Biomerieux Marcy l’Etoile, France). Conditioning of bioglass was obtained by incubation of test tubes for 48 hours at 37°C in order to promote the granules packaging. Value of pH was measured by pH meter at regular intervals to determine ions release and pH changing suggestive for conditioning.

#### Evaluation of antibacterial activity

In order to evaluate the antibacterial activity of BAG-S53P4, methicillin resistant *S. aureus* (MRSA) (n = 5), methicillin resistant *Staphylococcus epidermidis* (MRSE) (n = 5), *Pseudomonas aeruginosa* (n = 5) and *Acinetobacter baumannii* (n = 5) isolated from patients affected by chronic (> 6 months duration) osteomyelitis were considered. Two hundred microliters of a bacterial suspension (10^6^ CFU/mL) of each strain were inoculated into tubes containing BAG-S53P4 or inert glass. Tubes were incubated at 37°C in aerobic atmosphere and after 24, 48 and 72 hours of incubation 10 μL of proper diluted medium were plated on Trypticase Soy Agar plates for colonies counts. Values were expressed as log_10_CFU/mL. Tests were performed in duplicate.

### *In vivo* study

A prospective, cohort, observational study was performed from October 2010 to May 2013 on 27 patients (18 males, 9 females; mean age 44 ± 14 yrs, range 20–80 yrs) with a clinically and radiologically diagnosed osteomyelitis of the long bones and candidate to surgical debridement and defect filling with antibacterial bone substitute. All the patients gave their informed consent to participate in the study which was approved by the Ethics committee of our Institution and conducted in accordance with institutional standards.

Inclusion criteria were: age > 18 years, the presence of osteomyelitis of a long bone for at least 6 months requiring surgical debridement and bone defect filling; exclusion criteria were: need for local plastic procedures, segmental bone defects, associated septic arthritis.

Primary endpoint of the study was the absence of infection recurrence/persistence at follow-up, defined as: absence of draining sinus or local clinical signs of acute inflammation (redness, swelling, warmth, pain), elevated values of C reactive protein (CRP) and erythrocyte sedimentation rate (ESR) and no need for further surgery whenever during follow-up.

Secondary endpoint was the absence of local or systemic side effects connected with the use of the BAG.

#### Patients characteristics

Patients characteristics are reported in Table 1. Average symptoms duration at the time of surgery was 17.8 ± 14 months (range: 5–60 months). Mean previous surgeries before index operation were 1.8 ± 1.3 (range: 0–5). Pathogenesis of the infection was haematogenous in 8 patients, post-traumatic in 9 and post-surgical in 10. Infection was localized at the tibia in 18 cases, at the femur in 8, at the humerus or in the metatarsus in one patient each. One patient presented a bifocal localization (tibia and femur).

**Table 1:** Patients’ characteristics

**Patient**	**Age (years)**	**Sex**	**Site**	**Previous surgeries**	**Infection duration (months)**	**Pathogenesis**	**Stage**	**Host type**
1	35	F	Tibia	3	12	Post-traumatic	4	A
2	50	M	Femur	5	60	Post-traumatic	3	B (smoker)
3	60	M	Femur	2	18	Haematogenous	1	B (lymphoma, chemotherapy)
4	47	M	Tibia	4	54	Haematogenous	1	C (smoker, vasculopathy, diabetes)
5	35	M	Femur	1	18	Post-traumatic	3	A
6	23	M	Tibia	1	9	Post-surgical	3	A
7	80	M	Tibia	3	38	Post-surgical	3	B (psoriasis, corticosteroids)
8	41	M	Tibia	2	12	Post-surgical	3	A
9	38	M	Femur	1	36	Post-surgical	3	A
10	28	M	Femur	1	12	Post-surgical	3	A
11	40	F	Femur	1	12	Post-traumatic	3	B (tumor, radiotherapy)
12	50	F	Humerus	0	10	Haematogenous	1	B (rheumatoid arthritis)
13	40	F	Tibia	3	24	Post-traumatic	2	B ( peripheral vasculopathy)
14	60	M	Tibia	2	12	Post-traumatic	4	B (smoker)
15	44	M	Tibia	3	24	Post-traumatic	3	B (smoker, alcol abuse)
16	36	F	Tibia	0	6	Haematogenous	1	A
17	55	M	Tibia	2	12	Post-surgical	3	B (smoker, peripheral vasculopathy)
18	20	M	Tibia	0	6	Haematogenous	1	A
19	36	M	Tibia	2	6	Post-surgical	3	A
20	40	M	Femur	2	12	Post-surgical	3	B (smoker, peripheral vasculopathy)
21	30	M	Tibia	0	10	Haematogenous	1	B (tetraplegia, recurrent pneumonia and urinary tract infection)
22	50	F	Tibia	2	24	Post-traumatic	3	B (smoker)
23	52	F	Tibia	2	12	Haematogenous	1	B (diabetes, peripheral neuropathy)
24	50	M	Tibia	2	12	Post-surgical	3	B (Churg-Strauss disease, immunosuppression)
25	61	F	Foot	2	12	Post-surgical	3	B (diabetes)
26	60	M	Tibia	2	6	Post-traumatic	3	B (smoker)
27	20	F	Tibia + Femur	0	12	Haematogenous	1	B (immunosuppressive therapy)

According to Cierny & Mader anatomo-pathological classification [3], 8 patients were classified as Stage 1 osteomyelitis, 1 as Stage 2, 16 as Stage 3 and 2 as Stage 4. Host type was classified according to McPherson [4], 9 patients were considered Type A , 17 as Type B and 1 as Type C (Table 1).

All patients underwent pre-operative clinical and laboratory tests evaluation, x-ray, CT and MRI scan. Concerning clinical presentation and local inflammatory signs (redness, swelling, pain, local warmth), 8 patients were considered to have an acute infection presentation (all inflammatory signs present), 7 subacute (presence of at least two inflammatory signs) and 12 a chronic infection (presence of only one or no signs of local inflammation). Ten patients had a draining sinus at the time of surgery. Average pre-operative ESR and CRP values were 58 ± 41 mm/hr (range: 18 – 115 mm/hr) and 9.8 ± 7.3 mg/dL (range: 0.8 – 18 mg/dL), respectively.

#### Surgical procedure

Surgery was performed according to a same protocol in all the patients and by the same surgical team. In brief, with the patient laying supine, a surgical incision was made at the site of the osteomyelitic lesion, staying on previous scar, when present. After accurate dissection of soft tissues, bone was exposed. In some cases intra-operative fluoroscopy was used to localize the lesion. Removal of all foreign materials, when present (plates, screws, cerclages, bone substitutes, etc.), was then performed. Opening of the osteomyelitc focus was usually obtained with a surgical oscillating saw and osteotomes, in order to make a bone window of approximately 1 to 2 cm width and 2 to 8 cm length, depending on the infected site. Accurate debridement of the medullary canal was then performed with curettes, osteotomes and motorized burrs. Particular attention was paid to remove all necrotic tissues, sequestra and infected bone and soft tissues. Medullary canal proximal and distal to the lesion was opened. Repeated lavage with saline and accurate haemostasis was then performed. The use of tourniquet was avoided or restricted to a minimum.

After debridement and re-gowning and re-gloving, the bone defect was filled with BAG-S53P4 granules. No local antibiotics were added. Mean BAG volume used was 21.0 ± 10.9 mL (range: 2 – 60 mL). In two patients (1 and 14), classified as having a septic non-union, an external fixator was also implanted at the end of the surgical procedure.

All patients received thrombohaembolic prophylaxis with low weight heparin for four to six weeks and systemic antibiotic therapy with two antibiotics, targeted to the isolated microorganism(s) for four to six weeks post-operatively. In case of negative cultures a combination of two antibiotics was also empirically administered (usually intravenous vancomycin or teicoplanin and meropenem during hospital stay and then levofloxacin and rifampicin orally after discharge home).

#### Microbiological analysis

Microbiological analysis was conducted on the removed foreign material, when present, swabs and tissue samples (n = 5–8). Samples were aseptically collected at the site of surgery and delivered to the laboratory within 30 minutes.

Tissue samples and foreign materials were processed by sonication as routinely performed in our laboratory [20]. Briefly, the container was filled with sterile saline until complete submersion of the sample, carefully sealed and sonicated in an ultrasound bath (VWR, Milan, Italy) for 5 minutes with a frequency of 30 kHz and a power output of 300 W at room temperature. At the end of sonication, the obtained fluids were accurately mixed, centrifuged at 3000 rpm for 10 minutes and resuspended in a volume of about 1.5 mL.

Swabs and 100 μl of sonicated samples were seeded onto Chocolate Agar (CA), Mannitol Salt agar (MSA), MacConkey agar (MC) Schaedler Blood Agar (SBA), Sabouraud Agar (SA,) Brain Heart Infusion broth (BHI) and Thioglycollate Broth (TH). CA and MC plates were incubated at 37°C for 24 hours in 10% CO_2_ enriched atmosphere and aerobiosis, respectively. Incubation of MSA and SA lasted 48 hrs in aerobiosis while SBA was incubated in anaerobiosis at 37°C for 48 hours. After incubation, growth and colonies counts were recorded for both aerobes and anaerobes. BHI and TH were incubated for 15 days at 37°C and checked daily for microbial growth.

Identification was performed at biochemical (Vitek2 Compact, Biomerieux, Marcy l’Etoile, France) and genotypic level. Genotypical identification was performed by DNA sequencing of about 80 pb of variable regions V1 and V3 of the 16S rRNA gene by Pyrosequencing (PSQ96RA, Diatech, Jesi, Italy) [21]. Obtained sequences were inserted in BLAST (http://blast.ncbi.nlm.nih.gov/Blast.cgi) to perform accurate identification.

#### Post-surgical follow-up

All the patients underwent clinical and laboratory tests evaluation, including haemocromocitometric analysis with leukocyte formula and determination of ALT, AST, creatinine, CRP and ESR, at 15 and 30 days and at 3, 6, 9, 12, 18, 24 months after surgery.

X-ray examination was performed at 6, 12 and 24 months post-operatively.

Any early and late side effect presumably due to the local application of BAG were recorded at each visit.

#### Statistical analysis

Differences in colonies counts between BAG-S53P4 and inert glass were evaluated by means of two-way analysis of variance (ANOVA) and Bonferroni’s *t* test.

## Results

### *In vitro* study

During conditioning, a steady increase of pH toward basic values occurred in test tubes containing bioglass while no changes were observed for inert glass, which maintained a neutral pH. After 48 hours, pH values were 11.4 and 7.2 for BAG-S53P4 and inert glass, respectively, thus indicating occurrence of bioglass conditioning.

**Figure 1:**
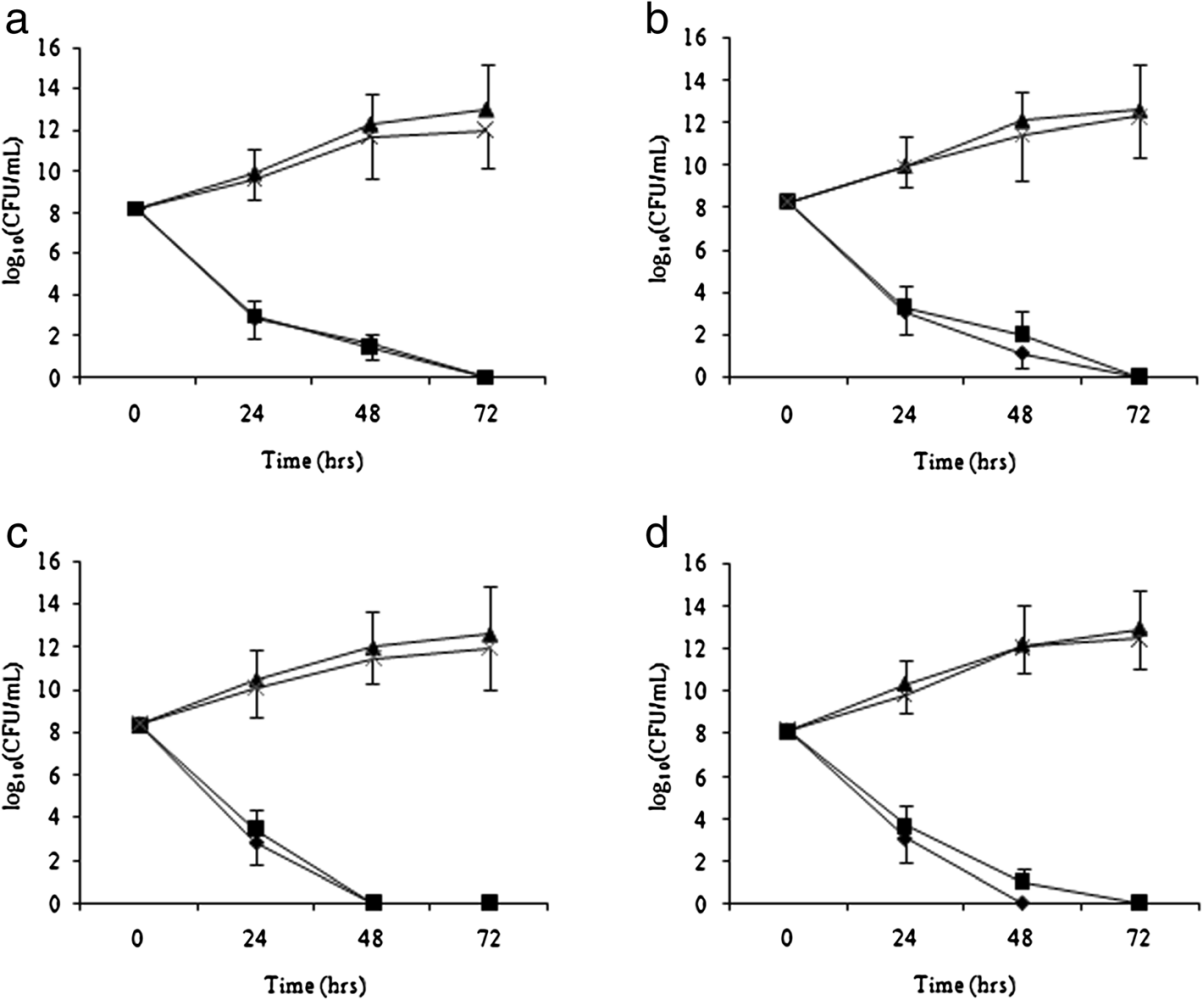
***In vitro *antibacterial activity of Bioglass S53P4 against methicillin-resistant *S. aureus *(a), *P. aeruginosa *(b), methicillin resistant *S. epidermidis *(c) and *A. baumanni *(d).** Data are mean ± S.D. of counts from 5 strains for each species. Circle: Bioglass S53P4 800 mg/mL; Square: Bioglass S53P4 400 mg/mL; Triangles: inert glass 800 mg/mL and Crosses: inert glass 400 mg/mL.

As shown in Figure 1, antimicrobial activity of the two different concentrations of conditioned BAG-S53P4 glass appears to be rather similar for all the tested strains.

After 24 hours of incubation with bioglass a significant decrease of about 5 logs of CFU/mL for all the tested species was observed. After 48 hours there was a further decrease in MRSA and *P. aeruginosa* colony counts while no bacterial growth was detected for MRSE and *A. baumannii*. After 72 hours of incubation a total absence of growth was observed for all clinical isolates. By contrast, all the tested strains showed an exponential increase in colony number in the tubes with inert glass. Statistical analysis evidenced significant differences *vs* time 0 and among treatment groups at all time tested with p values <0.002.

### *In vivo* study

Average hospital stay was 8.0 ± 6.9 days (range: 4–18 days). The isolated microorganisms are reported in Table 2. The most common isolated pathogen was *S. aureus* (18 patients, 10 with MRSA). In 5 patients a mixed flora was found, while in 4 patients cultures yielded negative results.

**Table 2:** Post-surgical data

**Patient**	**Microorganism**	**Follow-up (months)**	**Infection recurrence/persistance**
1	Multi-resistant *Enterococcus faecium*	30	None
2	MRSA	30	None
3	MRSA	26	None
4	MSSA	25	None
5	*Pseudomonas aeruginosa + Enterococcus faecium*	24	None
6	MRSA	24	None
7	*P. aeruginosa* + MSSA + *Enterococcus* spp.	24	Recurrence after 6 months
8	Negative	22	None
9	MSSA	20	None
10	MRSA	20	None
11	Negative	19	None
12	MSSA	18	None
13	*P. aeruginosa +* MSSA	17	Plastic flap after 8 months
14	MRSA + *Staphylococcus lugdunensis*	16	None
15	*Staphylococcus hominis*	16	None
16	MSSA	15	None
17	MSSE	15	None
18	MRSA + GABHS	14	None
19	Negative	14	None
20	MRSE	14	None
21	MRSA	13	None
22	MRSA	12	Recurrence after 6 months
23	MSSA	12	None
24	MRSA	12	None
25	Negative	10	None
26	MRSA	9	None
27	MSSA	9	None

*MRSA*: methicillin resistant *S. aureus*; *MSSA*: methicillin susceptible *S. aureus*; *GABHS*: Group A β-haemolytic streptococcus.

At a mean follow-up of 17.8 ± 6.1 months (range: 9 to 30 months), 24 patients (88.9%) did not demonstrate any sign of infection, while 2 showed an infection recurrence at 6 months from index operation and one more needed further surgical procedures (local muscular flap for delayed skin necrosis and bone exposure). Patient 21 died 13 months after surgery for the sequelae of recurrent pneumonia. No recurrence at the osteomyelitis site was evident at clinical or x-ray examination.

Patient 12 showed a prolonged (four weeks) serum wound leakage, requiring the application of three stitches and healed without further complications. The patient did not show any infection recurrence at the latest follow-up. No other side effects were recorded.

X-ray examination showed incorporation of the bioglass within the host bone and no signs of osteolysis or periosteal reactions. However, the biomaterial was still visible at x-ray examination at two years from surgery.

## Discussion

This is the first study reporting on the *in vitro* antibacterial effect of BAG-S53P4, compared to inert glass, against multi-resistant pathogen isolated in chronic osteomyelitis foci.

We also report the largest continuous series of patients affected by chronic osteomyelitis of the long bones, treated according to the same protocol, including surgical debridement and bone defect filling with BAG-S53P4, without any local antibiotic administration.

Until now autograft bone with its osteoinductive potential has been the material of choice when bone graft is needed [22]; but in the presence of infection it is considered contraindicated [23]. Many different methods have been used to treat the bone defect and the infection, including bone transport through an external fixator, surgical debridement and bone defect filling with antibiotic-impregnated polymethylmethacrylate (PMMA) or antibiotic-loaded bone grafts or bone substitutes, but clinical resolution still remains a challenge, while persistent serum wound leakage using calcium-based bone substitutes is a rather frequent occurrence [23].

Bioactive materials are defined as materials stimulating a specific biological response at the interface between the material and tissue, resulting in the formation of a bond between them [24]. In particular, bioactive glasses are bone substitutes with bone binding and antibacterial properties, first introduced in 1969 [24,25]. Since the degree of bone bonding capacity and resorption rates are highly affected by the chemical composition [26], evaluation of each bioglass is needed in order to improve their clinical use.

The present study had double aim: first to confirm, through *in vitro* tests*,* the antibacterial properties of BAG-S53P4 against multiresistant bacterial strains, isolated from patients with chronic osteomyelitis. Secondarily, to evaluate the *in vivo* efficacy and safety of BAG-S53P4 as a bone graft substitute in clinical setting by studying a continuous series of patients affected by chronic osteomyelitis of the long bones.

Results obtained in the first step of the study indicate a notable antibacterial activity of bioglass BAG-S53P4 granules at two different concentrations (800 mg/mL and 400 mg/mL) against methicillin resistant *S. aureus* and *S. epidermidis,* and multiresistant *P. aeruginosa* and *A. baumannii*. These results are in line and extend the antibacterial spectrum of activity of BAG-S53P4, previously reported [13,15,27]; to date, to our knowledge, no bacterial resistance to the action of this compound has been described.

Bioglass antibacterial property is probably due to the ability of granules to release ions, such as sodium, calcium, phosphate and silicate in aqueous conditions, which increase the pH value and the osmotic pressure of the environment [28]. For this reason, BAG-S53P4 granules need to be conditioned in order to create the right hostile environment for bacterial growth. In this study, *in vitro* conditioning was performed by leaving the glass in contact with an aqueous solution in order to activate the release of the ions responsible of raising the pH. The chosen conditioning time was 48 hours since no significant variations of pH were recorded if conditioning was further prolonged. Such a long time could be explained by the fact that in the experimental conditions used in this study, the contact surface between the aqueous environment and the bioglass was rather limited.

Furthermore bioactive glass has been proved to have angiogenic properties *in vitro*[10]. Vascularization and new bone formation have been observed to be faster in defects filled with BAG-S53P4 than in hydroxyapatite-filled defects. Initial fibrous tissue formation related to a considerable amount of blood vessels was also more rapid in the BAG filled defects [22,29]. Similar findings have been observed by Virolainen et al. who observed that the BAG surface is not only conductive but also osteoproductive in promoting migration, replication, and differentiation of osteogenic cells and their matrix production [30]. This phenomenon may be beneficial in treatment of osteomyelitis, as the antibacterial, osteoproductive and angiogenesis promoting properties observed for BAGs may remain over a long period.

The safety and efficacy of BAG-S53P4 have been clinically evaluated in randomized prospective clinical trials in the field of spine, benign bone tumour and trauma surgery and for the treatment of bone defects in oral and maxillo-facial surgery [31-34]. Despite the *in vitro* evidence of antibacterial efficacy of BAG-S53P4, a relatively limited number of clinical studies have explored the potential use of these materials for the treatment of chronic osteomyelitis. To our best knowledge, only two studies, on limited patients’ populations have been published so far concerning a possible application for the treatment of osteomyelitis of the long bones [14,19]. Our results confirm, on a larger population, previous studies, showing an approximately 90% eradication rate at an average 18 months follow-up, independently on the isolated pathogen. Of the two patients showing recurrent infection, one had a polymicrobial ethiology (*P. aeruginosa*, *S. aureus* and *Enterococcus* spp.), while in the other an MRSA was isolated at time of surgery. A third patient required a plastic procedure 8 months after bioglass implant and was considered a failure, according to success/failure criteria used in the present study. Further analysis of the failed cases suggests, for patient 7, an insufficient filling of the bone defect, since he had a long infected nail crossing both the tibia and femur, after a knee arthrodesis, that was removed by us at the time of debridement and bioglass application. Filling such a large defect was not feasible in this particular case. In a previous study on 11 patients, Lindfors and coauthors already hypothesized that outcome after BAG-S53P4 treatment might be related to proper filling of the cavities [14], and we think this was not achieved in this particular case.

An effective filling of large bone defects, consequent to infection bone matrix dissolution, debridement surgery and often associated to vascular defects, represents an important issue for bioactive materials since the dead space may easily harbor bacteria [33].

The other two failed patients shared soft tissue defects, that might have impaired the final outcome. Both of them were candidate to a simultaneous flap coverage at the time of debridement, that had not been undertaken because direct closure was finally achieved. In spite of this, both suffered healing wound problem after surgery and one of them finally received a fascio-cutaneous flap, with resolution of the septic process, while the other refuses further surgery, in the presence of an intermittently draining fistula.

## Conclusions

Following this experience, bioglass application is now recommended in our practice for those cases in which we may achieve a satisfactory filling of the defect and an adequate soft tissue coverage.

Limitations to the present study include the still relatively small limited population of patients and follow-up that, however, largely exceeded those previously published. Another limitation of the present study is the lack of a comparative series of patients, treated with other currently available surgical options, including surgical debridement and bone grafting or antibiotic-loaded bone substitutes.

In this regard it should be noted, however, that prospective comparative studies on different surgical treatments of osteomyelitis are extremely rare in the literature and difficult to perform, due to the high variability of the cases and to the challenging randomization process. For this reason in next future, it could be interesting to investigate the efficacy and safety of bioglass compared to two other retrospective case series, treated according to a different surgical protocol.

The *in vitro* and *in vivo* reported results do reinforce previous observations on the efficacy of BAG-S53P4 for the treatment of chronic osteomyelitis of the long bones, also in the presence of multi-resistant strains and in immunocompromised hosts, without relevant side effects and without the need for locally adding antibiotics.

### Ethics committee and informed consent

The informed consent of all human subjects who participated in this prospective observational investigation, was obtained after that the nature of the procedure and possible discomforts and risks, have been fully explained. Consent for publication of clinical personal details was obtained from all participants. Ethics Committee of IRCCS Galeazzi Institute, aware of the type of study, has approved and supported this research.

## Competing interests

All authors have no relevant conflicts of interest or financial conflicts to disclose. None of the authors have any relationship with the manufacturers of BAG-S53P4.

## Authors’ contributions

LD and CLR conceived the study, designed the experiments, analyzed data and revised the manuscript critically. CV and EDV performed *in vitro* experiments, analyzed data, wrote and revised the manuscript. CLR, NL and DR performed clinical study. RM revised the manuscript critically and partecipated in data analysis. All authors read and approved the final manuscript.

## Pre-publication history

The pre-publication history for this paper can be accessed here:

http://www.biomedcentral.com/1471-2334/13/584/prepub
